# Sarm1-mediated neurodegeneration within the enteric nervous system protects against local inflammation of the colon

**DOI:** 10.1007/s13238-021-00835-w

**Published:** 2021-04-19

**Authors:** Yue Sun, Qi Wang, Yi Wang, Wenran Ren, Ying Cao, Jiali Li, Xin Zhou, Wei Fu, Jing Yang

**Affiliations:** 1 State Key Laboratory of Membrane Biology, School of Life Sciences, Center for Life Sciences, Peking University, Beijing, 100871 China; 2 IDG/McGovern Institute for Brain Research, Peking University, Beijing, 100871 China; 3 Chinese Institute for Brain Research, Beijing, 102206 China; 4 Shenzhen Bay Laboratory, Institute of Molecular Physiology, Shenzhen, 518055 China; 5 Department of General Surgery, Peking University Third Hospital, Beijing, 100191 China; 6 School of Medicine, Tsinghua University, Beijing, 100084 China; 7 Kunming Institute of Zoology, Chinese Academy of Sciences, Kunming, 650223 China

**Keywords:** 3D imaging, enteric nervous system, axonal degeneration, neurodegeneration, catecholaminergic axons, Sarm1, colitis

## Abstract

Axonal degeneration is one of the key features of neurodegenerative disorders. In the canonical view, axonal degeneration destructs neural connections and promotes detrimental disease defects. Here, we assessed the enteric nervous system (ENS) of the mouse, non-human primate, and human by advanced 3D imaging. We observed the profound neurodegeneration of catecholaminergic axons in human colons with ulcerative colitis, and similarly, in mouse colons during acute dextran sulfate sodium-induced colitis. However, we unexpectedly revealed that blockage of such axonal degeneration by the *Sarm1* deletion in mice exacerbated the colitis condition. In contrast, pharmacologic ablation or chemogenetic inhibition of catecholaminergic axons suppressed the colon inflammation. We further showed that the catecholaminergic neurotransmitter norepinephrine exerted a pro-inflammatory function by enhancing the expression of IL-17 cytokines. Together, this study demonstrated that Sarm1-mediated neurodegeneration within the ENS mitigated local inflammation of the colon, uncovering a previously-unrecognized beneficial role of axonal degeneration in this disease context.

**Electronic supplementary material:**

The online version of this article (10.1007/s13238-021-00835-w) contains supplementary material, which is available to authorized users.

## INTRODUCTION

Axonal degeneration is one of the hallmarks of neurodegeneration. This pathological event commonly occurs in neurological disorders such as Alzheimer’s disease, Parkinson’s disease, amyotrophic lateral sclerosis, multiple sclerosis, glaucoma, chemotherapy-induced neuropathy, and traumatic injury (Wang et al., 2012; Neukomm and Freeman, 2014; Coleman and Hoke, 2020). In the canonical view, pathological axonal degeneration leads to disruption of neural connections and directly contributes to detrimental disease defects. Accordingly, extensive research efforts have been focused on the regulatory mechanisms of this neurodegenerative event in the hope of revealing new strategies to treat those dreadful diseases (Neukomm and Freeman, 2014; Coleman and Hoke, 2020; Figley and DiAntonio, 2020).

Significant advances in the knowledge of pathological axonal degeneration have been achieved in the past decades. The existence of specific molecular machinery for axonal degeneration was firstly revealed by the discovery of the Wallerian degeneration slow (*Wld*^*s*^) mutant mouse and the subsequent characterization of the Wld^s^ protein, whose gain-of-function prolonged the survival of traumatically-injured axons (Lunn et al., 1989; Mack et al., 2001). Notably, the protective action of the Wld^s^ protein prevailed in diverse neurodegenerative contexts, implicating that axonal degeneration could be instructed by convergent pathway(s) (Coleman, 2005; Coleman and Hoke, 2020). Recent studies showed that the genetic deletion of *Sarm1* (sterile alpha and HEAT/Armadillo motif containing 1) conferred robust axonal protection against traumatic injuries (Osterloh et al., 2012; Gerdts et al., 2013), as well as other pathological insults such as chemotherapy-induced neuropathy or mitochondrial poisoning (Yang et al., 2015; Geisler et al., 2016; Wang et al., 2019; Loreto et al., 2020). Moreover, our colleague and we have elucidated that axonal degeneration is intrinsically connected to the phenomenon of local energy deficit. The rapid depletion of axonal ATP is triggered under neurodegenerative conditions, which depends on the Sarm1 signal and would be inhibited by the Wld^s^ protein (Godzik and Coleman, 2015; Yang et al., 2015). In addition, the Sarm1 protein possesses the unique ability to degrade the metabolic coenzyme NAD^+^ (Essuman et al., 2017), which could be causative to the NAD^+^ loss observed during axonal degeneration (Wang et al., 2005). We also discovered that the high concentration of cellular NAD^+^ acts as an allosteric regulator to suppress the Sarm1 signal in healthy axons (Jiang et al., 2020).

Of importance, pathological axonal degeneration is distinct from several types of programmed cell death, e.g., apoptosis, necroptosis, or pyroptosis. For example, although the classic apoptotic pathway has essential roles in developmental axonal death, which occurs during the normal establishment of the nervous system (Luo and O’Leary, 2005), it is excluded from pathological scenarios of axonal degeneration (Whitmore et al., 2003; Simon et al., 2012). Conversely, the developmental death of neurons or axons is not affected by the Wld^s^ expression or the *Sarm1* deletion (Hoopfer et al., 2006; Osterloh et al., 2012).

With these research advances, a conceptual challenge has been realized in the field, i.e., why the body needs such signaling components designated for pathological axonal degeneration if their action is to enable neurodegenerative diseases? One plausible answer is that the timely destruction of traumatically-injured axons would facilitate axonal regeneration in the peripheral nervous system (Brown et al., 1992). However, besides such a few known examples, whether axonal degeneration might exert any beneficial role in a specific disease remains mostly unknown.

The enteric nervous system (ENS) is functionally separated from the central and peripheral nervous systems. It has indispensable roles in controlling different physiological aspects of the gastrointestinal tract (Goyal and Hirano, 1996; Furness, 2012). Although pathological alterations of the ENS have been reported (De Giorgio et al., 2004; Obermayr et al., 2013; Rao and Gershon, 2016), the occurrence and the underlying mechanism of the ENS neurodegeneration needs to be better characterized. In this study, we examined the ENS of the mouse, non-human primate, and human by advanced 3D imaging. We observed the profound neurodegeneration of catecholaminergic axons in human colons with ulcerative colitis, and similarly, in mouse colons during acute dextran sulfate sodium (DSS)-induced colitis. This neurodegenerative event was caused by TNFα and involved the local energy deficit. Unexpectedly, the blockage of such axonal degeneration by the *Sarm1* deletion in mice exacerbated the colitis condition. In contrast, pharmacologic ablation or chemogenetic inhibition of catecholaminergic axons markedly suppressed the colon inflammation. We went on to show that the loss of catecholaminergic axons depleted the neurotransmitter norepinephrine, which would otherwise promote the expression of pro-inflammatory IL-17 cytokines by T_h_17 and type 3 innate lymphoid cells (ILC3s). These findings demonstrate that Sarm1-mediated neurodegeneration within the ENS could mitigate local inflammation of the colon, revealing a previously-unrecognized beneficial role of axonal degeneration in this disease scenario.

## RESULTS

### 3D anatomy of the enteric nervous system of the mouse, non-human primate, and human

We sought to determine the 3D anatomy of the ENS in the intact, unsectioned gut tissues, of which only limited examples were reported (Neckel et al., 2016; Graham et al., 2020). We exploited the iDISCO(ace) method, an improved version of iDISCO+ that we developed with the acetone-based processing steps (Liu et al., 2020). iDISCO(ace) maintains the histologic integrity of tissues through the procedure of whole-tissue immunolabeling and optical clearing and exhibits superior antibody compatibility, which is essential for the detection and visualization of diverse cellular structures.

**Figure 1:**
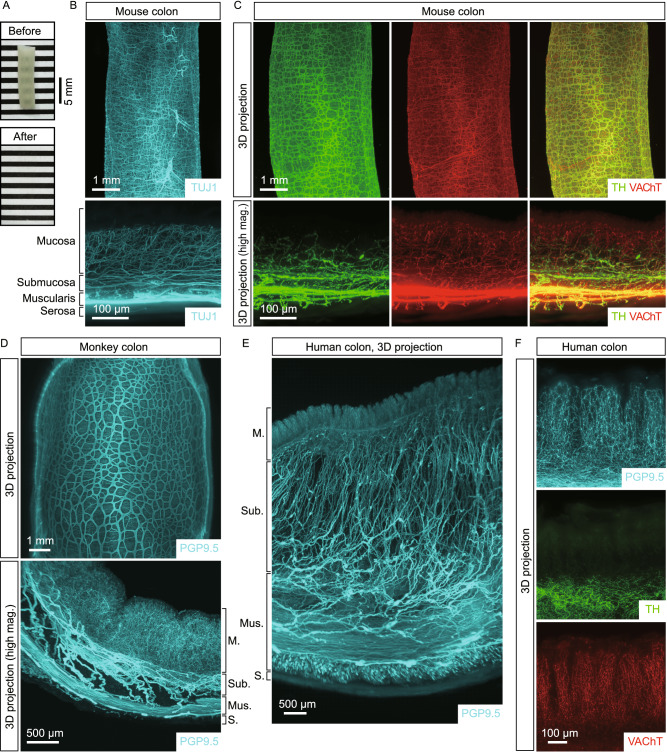
**3D anatomy of the enteric nervous system of the mouse, non-human primate, and human**. (A–C) 3D assessment of the ENS in the mouse colon. (A) The unsectioned colon tissue of the adult mouse before and after the iDISCO(ace) procedure. (B and C) The unsectioned colon tissues of adult mice were processed for the whole-tissue immunolabeling of TUJ1 (B) or the co-immunolabeling of TH and VAChT (C). Representative 3D-projection images at 1.26× magnification (upper panels) and 3D-projection images (longitudinal view) of the 500-μm thickness of the tissues at 12.6× magnification (lower panels) of the lightsheet imaging were shown. (D) 3D assessment of the ENS in the non-human primate colon. The unsectioned colon tissue of the adult macaque monkey was processed for the PGP9.5-immunolabeling. Representative 3D-projection image at 1.26× magnification (upper panel) and 3D-projection image (cross-sectional view) of the 2-mm thickness of the tissue at 6.4× magnification (lower panel) of the lightsheet imaging were shown. (E and F) 3D assessment of the ENS in the human colon. The unsectioned colon tissues of adult humans were processed for the whole-tissue immunolabeling of PGP9.5, TH, or VAChT. (E) Representative 3D-projection image (cross-sectional view) of the 2-mm thickness of the tissue at 1.26× magnification of the lightsheet imaging. (F) Representative 3D-projection images (cross-sectional view) of the 1-mm thickness of the mucosa at 12.6× magnification of the lightsheet imaging. The anatomical layers of the colon [mucosa (M.), submucosa (Sub.), muscularis (Mus.), and serosa (S.)] were indicated

We first examined the ENS in the gut tissues of adult mice. The iDISCO(ace) method rendered the unsectioned tissues completely transparent (Fig. 1A), facilitating the lightsheet imaging that we utilized throughout the study. The whole-tissue immunolabeling of TUJ1 (neuronal class III β-tubulin), a common pan-neural marker, revealed the intricate, mesh-like architecture of the ENS within the mouse colon (Fig. 1B). The high-magnification view showed the 3D distribution of neural organizations in the anatomical layers, i.e., Auerbach’s plexus in the muscularis, Meissner’s plexus in the submucosa, and dense innervations within the mucosa (Fig. 1B). Further, the co-immunolabeling of TH (tyrosine hydroxylase) and VAChT (vesicular acetylcholine transporter), the specific catecholaminergic and cholinergic markers, respectively, showed the 3D anatomy of these two major types of neural innervations in the colon (Fig. 1C and Video S1). Notably, catecholaminergic axons in the colon mucosa were predominantly present in the lamina propria and muscularis mucosae but mostly excluded from the epithelium (Fig. 1C). Similarly, the 3D distribution of catecholaminergic and cholinergic innervations in the mouse small intestine was visualized by the co-immunolabeling of TH and VAChT (Fig. S1D). In addition to the ENS, different cellular structures within the mouse gut could be imaged by iDISCO(ace), e.g., the vascular network by the immunolabeling of PECAM1 (platelet endothelial cell adhesion molecule 1) (Fig. S1A), the lymphatic network by the immunolabeling of VEGFR3 (vascular endothelial growth factor receptor 3) or LYVE1 (lymphatic vessel endothelial hyaluronic acid receptor 1) (Fig. S1B and S1E), and glial cells by the immunolabeling of GFAP (glial fibrillary acidic protein) (Fig. S1C). Also, the spatial correlation between immune cells, e.g., T cells, and neural innervations was exemplified by the co-immunolabeling of CD3 together with TH and VAChT (Video S2).

Next, we pursued the 3D anatomy of the primate ENS. The unsectioned gut tissues of a non-human primate, i.e., rhesus macaque monkey, were processed for the whole-tissue immunolabeling of PGP9.5 (protein gene product 9.5), another specific pan-neural marker. The mesh-like 3D architecture of the ENS in the monkey colon was revealed (Fig. 1D), which resembled that of the mouse ENS. The high-magnification views exhibited the neural organizations in the different anatomical layers of the colon and small intestine (Figs. 1D and S1F). Moreover, we examined the unsectioned human gut tissues. Despite the significantly thickened muscularis and submucosa of the human colon, the PGP9.5-immunolabeling showed the preservation of the 3D architecture of neural innervations (Fig. 1E). Also, the TH-immunolabeling confirmed the distinct pattern of catecholaminergic axons within the colon mucosa, i.e., the dense presence in the lamina propria and muscularis mucosae but not in the epithelium (Fig. 1F). As well, the 3D distribution of neural innervations in the human small intestine was elucidated by the PGP9.5-immunolabeling (Fig. S1G). Together, these results documented the 3D anatomy of the ENS of the mouse, non-human primate, and human.

### Neurodegeneration of catecholaminergic axons within the human or mouse ENS under colitis conditions

**Figure 2:**
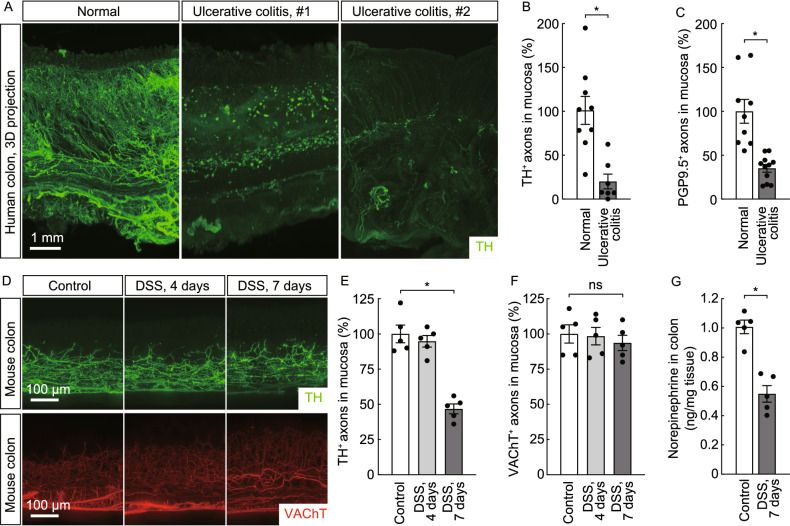
**Neurodegeneration of catecholaminergic axons within the human or mouse ENS under colitis conditions**. (A–C) Neurodegeneration of catecholaminergic axons in the human colon with ulcerative colitis. (A) The normal human colon tissue (left panel) or colon tissues of two patients with ulcerative colitis (right panels) were processed for the whole-tissue TH-immunolabeling. Representative 3D-projection images (cross-sectional view) of the 2-mm thickness of the tissues at 1.26× magnification of the lightsheet imaging were shown. (B) The human colon tissues under normal condition (*n* = 9) or with ulcerative colitis (*n* = 7) were processed for conventional anti-TH immunohistochemistry. TH-positive catecholaminergic axons within the mucosa were quantified. Mean ± SEM, *P < 0.01 (Student’s *t*-test). (C) The human colon tissues under normal condition (*n* = 9) or with ulcerative colitis (*n* = 11) were processed for conventional anti-PGP9.5 immunohistochemistry. PGP9.5-positive total axons within the mucosa were quantified. mean ± SEM, *P < 0.01 (Student’s *t*-test). (D–G) Neurodegeneration of catecholaminergic axons in the mouse colons during acute colitis. The wild-type mice were subjected to the DSS-induced colitis. (D–F) The unsectioned colon tissues were processed for the whole-tissue immunolabeling of TH or VAChT. (D) Representative 3D-projection images (longitudinal view) of the 500-μm thickness of the tissues at 12.6× magnification of the lightsheet imaging. (E and F) TH-positive catecholaminergic axons (E) or VAChT-positive cholinergic axons (F) within the mucosa were quantified. *n* = 5, mean ± SEM, *P < 0.01; ns, not significant (ANOVA test). (G) The norepinephrine content in the colon tissues was measured. *n* = 5, mean ± SEM, *P < 0.01 (Student’s *t*-test)

Aided with this 3D imaging power, we assessed the status of the ENS in the colon tissues of human patients with ulcerative colitis, a common type of inflammatory bowel disease. Strikingly, the profound neurodegeneration of catecholaminergic axons was observed by the whole-tissue TH-immunolabeling (Fig. 2A). The quantification revealed that over 80% of TH-positive catecholaminergic axons within the colon mucosa were lost under this colitis condition (Fig. 2B). Accordingly, there was also an approximately 60% decrease of PGP9.5-positive total axons (Fig. 2C).

With this observation in the human colitis, we examined the ENS in the mouse colon during DSS-induced acute colitis, a standard model of inflammatory bowel disease (Chassaing et al., 2014; Kiesler et al., 2015). Resembling that occurring in the human ulcerative colitis, the neurodegeneration of catecholaminergic axons was evident in the DSS-insulted mouse colons, with signs of axonal fragmentation and debris in the mucosa (Fig. 2D and 2E). In contrast, no significant loss of cholinergic axons was detected by the VAChT-immunolabeling (Fig. 2D and 2F). Notably, the cell bodies of catecholaminergic neurons of the celiac ganglia, which contribute to the majority of catecholaminergic innervations within the mouse colon, remained intact after the DSS treatment (Fig. S2A and S2B).

**Figure 3:**
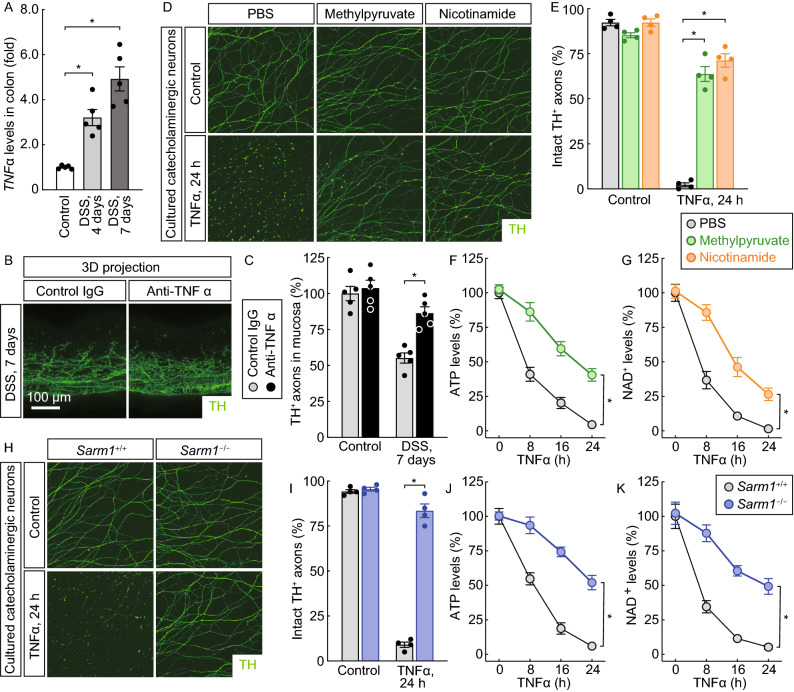
**Neurodegeneration of catecholaminergic axons involved the local energy deficit and depended on Sarm1**. (A–C) The degeneration of catecholaminergic axons in the colon was caused by TNFα. (A) The wild-type mice were subjected to the DSS-induced colitis. *TNF*α mRNA levels in the colon tissues were determined by the qPCR analysis. *n* = 5, mean ± SEM, *P < 0.01 (ANOVA test). (B and C) The wild-type mice were administered with the anti-TNFα neutralizing antibody or control IgG and then subjected to the DSS-induced colitis. The unsectioned colon tissues were processed for the whole-tissue TH-immunolabeling. (B) Representative 3D-projection images (longitudinal view) of the 500-μm thickness of the tissues at 12.6× magnification of the lightsheet imaging. (C) TH-positive catecholaminergic axons within the mucosa were quantified. *n* = 5, mean ± SEM, *P < 0.01 (ANOVA test). (D–G) TNFα directly triggered the axonal degeneration of catecholaminergic neurons of the celiac ganglia. Catecholaminergic neurons of the celiac ganglia of wild-type mice were *in vitro* cultured. The neurons were then treated with 50 ng/mL recombinant TNFα, in combination with 10 mmol/L methylpyruvate or 50 mmol/L nicotinamide. (D) Representative images of TH-positive axons of the cultured catecholaminergic neurons. (E) The integrity of TH-positive catecholaminergic axons was quantified. *n* = 4, mean ± SEM, *P < 0.01 (ANOVA test). (F and G) ATP (F) or NAD^+^ (G) levels of the catecholaminergic neurons were measured. *n* = 4, mean ± SEM, *P < 0.01 (ANOVA test). (H to K) TNFα-triggered axonal degeneration of catecholaminergic neurons of the celiac ganglia depended on Sarm1. Catecholaminergic neurons of the celiac ganglia of *Sarm1*^*+/+*^ or *Sarm1*^*-/-*^ mice were *in vitro* cultured and then treated with 50 ng/mL recombinant TNFα. (H) Representative images of TH-positive axons of the cultured catecholaminergic neurons. (I) The integrity of TH-positive catecholaminergic axons was quantified. *n* = 4, mean ± SEM, *P < 0.01 (ANOVA test). (J and K) ATP (J) or NAD^+^ (K) levels of the catecholaminergic neurons were measured. *n* = 4, mean ± SEM, *P < 0.01 (ANOVA test)

We set out to investigate this unique event of neurodegeneration within the ENS. Several pro-inflammatory cytokines, e.g., TNFα, IL-1β, IL-6, and IL-17A, are known to be involved in DSS-induced colitis. We tested whether such pro-inflammatory factors might directly trigger the degeneration of catecholaminergic axons. TNFα, whose expression levels were highly up-regulated in the DSS-insulted colon tissues (Fig. 3A), caused the massive axonal degeneration of *in vitro* cultured catecholaminergic neurons (Fig. 3D and 3E). On the other hand, the recombinant proteins of IL-1β, IL-6, and IL-17A did not significantly affect these axons (Fig. S2C and S2D). Moreover, the administration of an anti-TNFα neutralizing antibody was sufficient to preserve the catecholaminergic axons in the colon tissues after the DSS treatment (Fig. 3B and 3C), supporting the causative role of TNFα in this axonal degeneration within the ENS.

**Figure 4:**
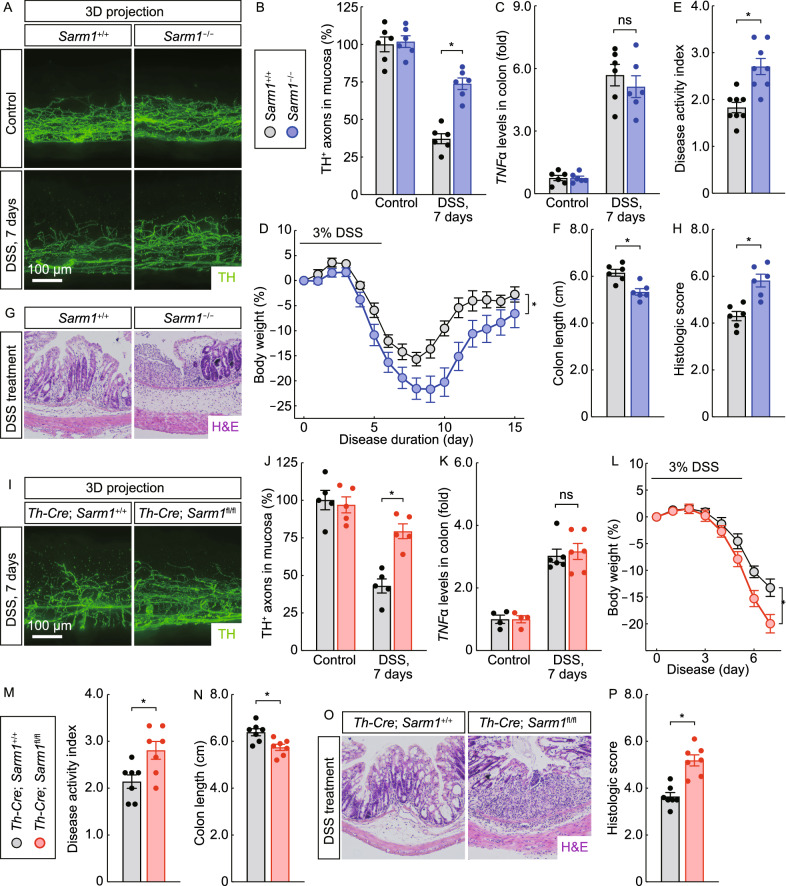
**Sarm1-mediated neurodegeneration of catecholaminergic axons protects against colitis**. (A–H) The blockage of pathological degeneration of catecholaminergic axons exacerbated the colitis condition. *Sarm1*^+/+^ or *Sarm1*^−/−^ mice were subjected to the DSS-induced colitis. (A and B) The unsectioned colon tissues were processed for the TH-immunolabeling. (A) Representative 3D-projection images (longitudinal view) of the 500-μm thickness of the tissues at 12.6× magnification of the lightsheet imaging. (B) TH-positive catecholaminergic axons within the mucosa were quantified. *n* = 6, mean ± SEM, *P < 0.01 (ANOVA test). (C) *TNF*α mRNA levels in the colon tissues were determined by the qPCR analysis. *n* = 6, mean ± SEM, ns, not significant (ANOVA test). (D) The body weights of the mice were monitored through the disease. *n* = 10, mean ± SEM, *P < 0.01 (ANOVA test). (E) The disease activity indexes of the mice at 7 days after the DSS treatment. *n* = 8, mean ± SEM, *P < 0.01 (Student’s *t*-test). (F) The colon lengths at 7 days after the DSS treatment. *n* = 6, mean ± SEM, *P < 0.01 (Student’s *t*-test). (G and H) The colon tissues at 7 days after the DSS treatment were processed for the H&E (hematoxylin and eosin) staining. (G) Representative images of the H&E-stained colon sections. (H) Histologic scores were determined. *n* = 6, mean ± SEM, *P < 0.01 (Student’s *t*-test). (I to P) The specific deletion of *Sarm1* in catecholaminergic neurons promoted the colitis condition. *Th-Cre*; *Sarm1*^+/+^ and *Th-Cre*; *Sarm1*^*fl*/*fl*^ mice were subjected to the DSS-induced colitis. (I and J) The unsectioned colon tissues were processed for the TH-immunolabeling. (I) Representative 3D-projection images (longitudinal view) of the 500-μm thickness of the tissues at 12.6× magnification of the lightsheet imaging. (J) TH-positive catecholaminergic axons within the mucosa were quantified. *n* = 5, mean ± SEM, *P < 0.01 (ANOVA test). (K) *TNF*α mRNA levels in the colon tissues were determined by the qPCR analysis. *n* = 6, mean ± SEM, ns, not significant (ANOVA test). (L) The body weights of the mice were followed through the disease. *n* = 7, mean ± SEM, *P < 0.01 (ANOVA test). (M) The disease activity indexes of the mice at 7 days after the DSS treatment. *n* = 7, mean ± SEM, *P < 0.01 (Student’s *t*-test). (N) The colon lengths at 7 days after the DSS treatment. *n* = 7, mean ± SEM, *P < 0.01 (Student’s *t*-test). (O and P) The colon tissues at 7 days after the DSS treatment were processed for the H&E staining. (O) Representative images of the H&E-stained colon sections (P) Histologic scores were determined. *n* = 7, mean ± SEM, *P < 0.01 (Student’s *t*-test)

Previous studies by our colleagues and us have demonstrated the energy deficit as the key feature of pathological axonal degeneration (Wang et al., 2005; Gerdts et al., 2015; Yang et al., 2015; Coleman and Hoke, 2020; Figley and DiAntonio, 2020). We observed that the depletion of ATP levels occurred in the cultured catecholaminergic neurons after the TNFα treatment (Fig. 3F). The cell-permeable form of pyruvate, i.e., methylpyruvate, delayed not only this ATP depletion but also the degeneration of catecholaminergic axons (Fig. 3D–F). At the same time, NAD^+^ levels decreased in these cultured neurons (Fig. 3G), which could be rescued by the exogenous supplement of the NAD^+^ metabolic precursor nicotinamide (Fig. 3D, 3E, and 3G). Furthermore, we explored whether this TNFα-triggered axonal loss would depend on Sarm1, the central regulator of pathological axonal degeneration. Indeed, the *Sarm1* deletion strongly suppressed the axonal degeneration of cultured catecholaminergic neurons (Fig. 3H and 3I), as well as the depletion of ATP and NAD^+^ levels upon the TNFα treatment (Fig. 3J and 3K). In addition, the degeneration of catecholaminergic axons within the mucosa was blocked in the DSS-insulted colon tissues of *Sarm1*^−/−^ mice (Fig. 4A and 4B), though *TNFα* mRNA levels were unaffected (Fig. 4C). Together, these results showed that this neurodegeneration of catecholaminergic axons involves the energy deficit and is controlled by the Sarm1 signal.

### Sarm1-mediated neurodegeneration of catecholaminergic axons protects against colitis

Despite its positive effect of blocking the pathological degeneration of catecholaminergic axons, we were surprised to observe that the *Sarm1* deletion significantly exacerbated the colitis condition, as evidenced by the worsened body-weight loss and disease activity index (Fig. 4D and 4E). Also, the tissue pathology, e.g., the shortened colon length, crypt loss, and immune cell infiltration, became more severe in *Sarm1*^−/−^ mice after the DSS treatment (Fig. 4F–H). Notably, although Sarm1 is predominantly expressed in neurons, it is also detectable in several populations of immune cells (Kim et al., 2007). In light of this unexpected disease-promoting outcome of the *Sarm1* deletion, we needed to distinguish its neuron-specific action. First, we utilized the approach of bone-marrow chimeric mice (BMCMs). The chimeric efficiency in BMCMs was over 90%, as assessed by the presence of CD45.1^+^-donor cells in the CD45.2^+^-recipient mice (Fig. S3A). However, the *Sarm1* depletion in immune cells did not affect the degeneration of catecholaminergic axons in the DSS-insulted colon tissues of *Sarm1*^−/−^-BMCMs (Fig. S3B and S3C). In addition, *Sarm1*^+/+^-BMCMs and *Sarm1*^−/−^-BMCMs underwent a similar course of colitis, as monitored by the daily change of body weight (Fig. S3D), suggesting that Sarm1 expressed in immune cells would likely not be involved. Next, we generated *Sarm1*^*fl*/*fl*^ mice to further prove the neuron-specific function of Sarm1 in this disease context (Fig. S3E). *Th-Cre*; *Sarm1*^*fl*/*fl*^ mice were bred to enable the specific deletion of *Sarm1* in catecholaminergic neurons. As expected, the degeneration of catecholaminergic axons after the DSS treatment was significantly inhibited in *Th-Cre*; *Sarm1*^*fl*/*fl*^ mice (Fig. 4I and 4J), although *TNFα* mRNA levels were comparable in the colon tissues of *Th-Cre*; *Sarm1*^+/+^ and *Th-Cre*; *Sarm1*^*fl*/*fl*^ mice (Fig. 4K). Moreover, similar to that shown above in *Sarm1*^−/−^ mice, *Th-Cre*; *Sarm1*^*fl*/*fl*^ mice had the exacerbated disease according to the body-weight loss and disease activity index (Fig. 4L and 4M). As well, the DSS-induced pathology was worsened in the colon tissues of *Th-Cre*; *Sarm1*^*fl*/*fl*^ mice (Fig. 4N–P). These results together supported that the Sarm1-mediated neurodegeneration of catecholaminergic axons within the colon protected against colitis.

### The catecholaminergic signal promotes local inflammation of the colon

**Figure 5:**
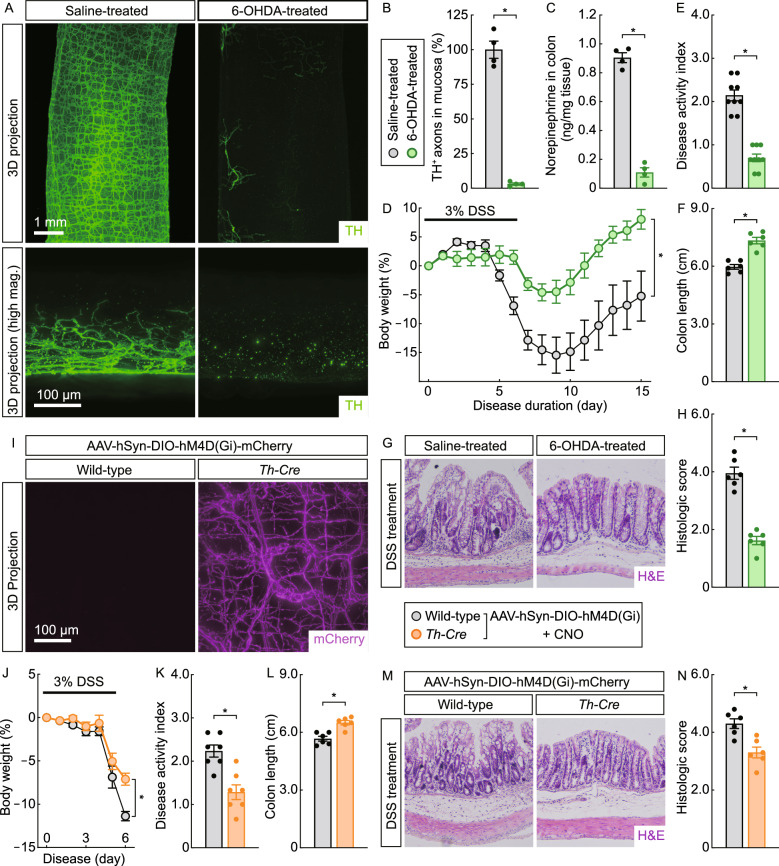
**Loss of the catecholaminergic signal mitigates the colitis condition**. (A–H) Pharmacologic ablation of catecholaminergic axons suppressed colitis. The wild-type mice were treated with 6-OHDA via intraperitoneal injection and then subjected to the DSS-induced colitis. (A and B) The unsectioned colon tissues were processed for the whole-tissue TH-immunolabeling. (A) Representative 3D-projection images of the tissues at 1.26× magnification (upper panels) and 3D-projection images (longitudinal view) of the 500-μm thickness of the tissues at 12.6× magnification (lower panels) of the lightsheet imaging were shown. (B) TH-positive catecholaminergic axons within the mucosa were quantified. *n* = 4, mean ± SEM, *P < 0.01 (Student’s *t*-test). (C) The norepinephrine content in the colon tissues was measured. *n* = 4, mean ± SEM, *P < 0.01 (Student’s *t*-test). (D) The body weights of the mice were monitored through the colitis condition. *n* = 10, mean ± SEM, *P < 0.01 (ANOVA test). (E) The disease activity indexes of the mice at 7 days after the DSS treatment. *n* = 9, mean ± SEM, *P < 0.01 (Student’s *t*-test). (F) The colon lengths at 7 days after the DSS treatment. *n* = 6, mean ± SEM, *P < 0.01 (Student’s *t*-test). (G and H) The colon tissues at 7 days after the DSS treatment were processed for the H&E staining. (G) Representative images of the H&E-stained colon sections. (H) Histologic scores were determined. *n* = 6, mean ± SEM, *P < 0.01 (Student’s *t*-test). (I to N) Chemogenetic inhibition of catecholaminergic axons alleviated the colitis condition. *Th-Cre* or wild-type mice were intraperitoneally injected with AAV viral vectors expressing the chemogenetic inhibitor [AAV-hSyn-DIO-hM4D(Gi)-mCherry]. The mice were then subjected to the DSS-induced colitis in combination with the CNO treatment. (I) The unsectioned colon tissues were processed for the whole-tissue mCherry-immunolabeling. Representative 3D-projection images (serosa-to-mucosa view) of the tissues at 12.6× magnification of the lightsheet imaging were shown. (J) The body weights of the mice were followed through the disease. *n* = 7, mean ± SEM, *P < 0.01 (ANOVA test). (K) The disease activity indexes of the mice at 7 days after the DSS treatment. *n* = 7, mean ± SEM, *P < 0.01 (Student’s *t*-test). (L) The colon lengths at 7 days after the DSS treatment. *n* = 6, mean ± SEM, *P < 0.01 (Student’s *t*-test). (M and N) The colon tissues at 7 days after the DSS treatment were processed for the H&E staining. (M) Representative images of the H&E-stained colon sections. (N) Histologic scores were determined. *n* = 6, mean ± SEM, *P < 0.01 (Student’s *t*-test)

We went on to verify this novel, disease-mitigating action afforded by the pathological degeneration of catecholaminergic axons. 6-OHDA could cause the axonal degeneration of cultured catecholaminergic neurons (Fig. S4A), which involved the local energy deficit and depended on the Sarm1 signal (Fig. S4A–D). We thus purposely induced the degeneration of catecholaminergic axons within the ENS via the intraperitoneal administration of 6-OHDA. The whole-tissue TH-immunolabeling confirmed the efficiency of this pharmacologic ablation in the colon (Fig. 5A and 5B). On the other hand, cholinergic axons were left intact in the 6-OHDA-treated colon tissues, as assessed by the VAChT-immunolabeling (Fig. S4E and S4F). Importantly, the pharmacologic ablation of catecholaminergic axons rendered the mice highly resistant to colitis with the milder body-weight loss and disease activity index (Fig. 5D and 5E). In addition, the DSS-induced pathology became alleviated in the colon tissues of 6-OHDA-treated mice (Fig. 5F–H), showing that the degeneration of catecholaminergic axons within the ENS countered the colitis condition.

We further validated that this protective effect against colitis resulted from the loss of the catecholaminergic signal. To this end, we exploited AAV viral vectors [AAV-hSyn-DIO-hM4D(Gi)-mCherry] that expressed the chemogenetic inhibitor hM4D(Gi) in the neuron-specific and Cre-dependent manner. The intraperitoneal injection of AAV viral vectors gave rise to the robust labeling of the colon tissues of *Th-Cre* mice but not those of wild-type mice, as visualized by the whole-tissue mCherry-immunolabeling (Fig. 5I). The wild-type mice that received the injection of AAV viral vectors exhibited the regular disease aspects following the DSS treatment in the presence of clozapine N-oxide (CNO) (Fig. 5J–N), ruling out any potential non-specific effect of AAV viral vectors or CNO. We found out that the chemogenetic inhibition of catecholaminergic axons in *Th-Cre* mice was sufficient to diminish the DSS-induced colitis, as examined by the body-weight loss and disease activity index (Fig. 5J and 5K). Also, this chemogenetic inhibition lessened the pathology in the DSS-insulted colon tissues of *Th-Cre* mice (Fig. 5L–N). These results proved that the loss of the catecholaminergic signal within the colon could mitigate colitis.

**Figure 6:**
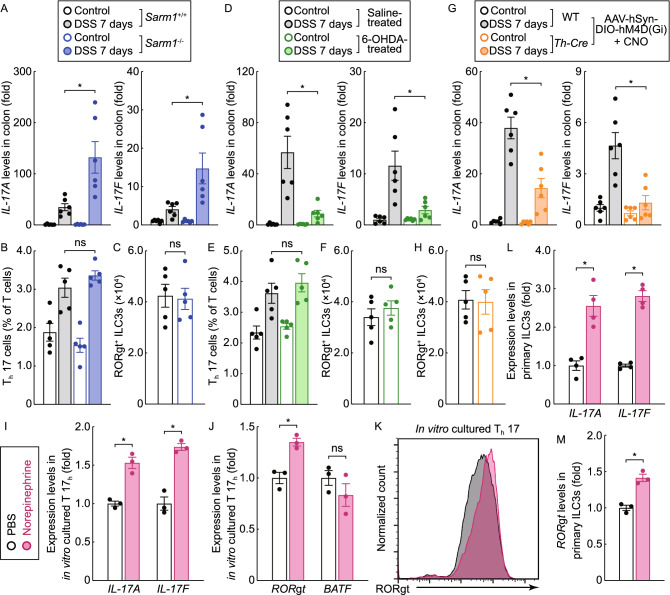
**Norepinephrine promotes the expression of pro-inflammatory IL-17 cytokines in colitis**. (A–C) The blockage of pathological degeneration of catecholaminergic axons enhanced the expression of IL-17 cytokines. *Sarm1*^+/+^ or *Sarm1*^−/−^ mice were subjected to the DSS-induced colitis. (A) *IL-17A* and *IL-17F* mRNA levels in the colon tissues were determined by the qPCR analysis. *n* = 6, mean ± SEM, *P < 0.01 (ANOVA test). (B and C) Foxp3^−^ RORγt^+^ T_h_17 cells (B) or RORγt^+^ ILC3s (C) in the colon tissues were FACS analyzed. *n* = 5, mean ± SEM, ns, not significant (ANOVA test). (D to F) Pharmacologic ablation of catecholaminergic axons suppressed the expression of IL-17 cytokines. The wild-type mice were treated with 6-OHDA via intraperitoneal injection and then subjected to the DSS-induced colitis. (D) *IL-17A* and *IL-17F* mRNA levels in the colon tissues were determined by the qPCR analysis. *n* = 6, mean ± SEM, *P < 0.01 (ANOVA test). (E and F) Foxp3^−^ RORγt^+^ T_h_17 cells (E) or RORγt^+^ ILC3s (F) in the colon tissues were FACS analyzed. *n* = 5, mean ± SEM, ns, not significant (ANOVA test). (G and H) Chemogenetic inhibition of catecholaminergic axons dampened the expression of IL-17 cytokines. *Th-Cre* or wild-type mice were intraperitoneally injected with AAV viral vectors expressing the chemogenetic inhibitor. The mice were then subjected to the DSS-induced colitis in combination with the CNO treatment. (G) *IL-17A* and *IL-17F* mRNA levels in the colon tissues were determined by the qPCR analysis. *n* = 6, mean ± SEM, *P < 0.01 (ANOVA test). (H) RORγt^+^ ILC3s in the colon tissues were FACS analyzed. *n* = 5, mean ± SEM, ns, not significant (ANOVA test). (I to K) Norepinephrine promoted the expression of IL-17 cytokines by T_h_17 cells. *In vitro* cultured T_h_17 cells were treated with 10 μmol/L norepinephrine. (I and J) mRNA levels of *IL-17A* and *IL-17F* (I) or *RORγt* and *BATF* (J) in T_h_17 cells were determined by the qPCR analysis. *n* = 3, mean ± SEM, *P < 0.01; ns, not significant (ANOVA test). (K) Protein levels of RORγt in T_h_17 cells were FACS analyzed. (L and M) Norepinephrine enhanced the expression of IL-17 cytokines by ILC3s. Primary ILC3s FACS-sorted from the colon tissues were *in vitro* treated with 10 μmol/L norepinephrine. mRNA levels of *IL-17A* and *IL-17F* (L) or *RORγt* (M) in ILC3s were determined by the qPCR analysis. *n* = 3, mean ± SEM, *P < 0.01 (ANOVA test)

We then looked into the mechanism underlying this beneficial action of pathological degeneration of catecholaminergic axons. The tissue content of the catecholaminergic neurotransmitter norepinephrine decreased in the colon tissues of wild-type mice after the DSS treatment (Fig. 2G), and such norepinephrine depletion was even more pronounced in those of 6-OHDA-treated mice (Fig. 5C). IL-17A and IL-17F have been established as the central pro-inflammatory cytokines in colitis (Iwakura et al., 2011; Miossec and Kolls, 2012), though a controversy regarding IL-17A is noted (Ito et al., 2008; Yang et al., 2008; Tang et al., 2018). We observed that the blockage of the degeneration of catecholaminergic axons by the *Sarm1* deletion resulted in the higher expression of *IL-17A* and *IL-17F* in the DSS-insulted colon tissues (Fig. 6A). Conversely, *IL-17A* and *IL-17F* levels were suppressed by pharmacologic ablation (Fig. 6D) or chemogenetic inhibition (Fig. 6G) of catecholaminergic axons. These observations suggested the possibility that norepinephrine might promote the expression of these two pro-inflammatory cytokines.

Therefore, we examined T_h_17 cells, the predominant producer of IL-17A and IL-17F in tissues. The presence of T_h_17 cells in the colon tissues was comparable between *Sarm1*^+/+^ or *Sarm1*^−/−^ mice (Fig. 6B). Also, Foxp3^+^ RORγt^−^ or Foxp3^+^ RORγt^+^ regulatory T cells were not altered in *Sarm1*^−/−^ mice (Fig. S5A). Similarly, the pharmacologic ablation of catecholaminergic axons did not affect the percentage of T_h_17 cells or regulatory T cells in the colon tissues (Figs. 6E and S5C). In addition, these two different manipulations of catecholaminergic axons showed no effect on the mRNA levels of *IL-23* (Fig. S5B and S5D), a cytokine critical for the function of T_h_17 cells. However, we discovered that in the cultured T_h_17 cells (Fig. S5E), norepinephrine effectively elevated *IL-17A* and *IL-17F* mRNA levels (Fig. 6I). Moreover, the transcription factor RORγt, which is essential for the IL-17 expression (Ivanov et al., 2006), was significantly enhanced in T_h_17 cells by the norepinephrine treatment (Fig. 6J and 6K). On the contrary, norepinephrine did not increase the expression of *BATF* (Fig. 6J), another transcription factor critical for T_h_17 cells (Schraml et al., 2009).

In addition to T_h_17 cells, type 3 innate lymphoid cells (ILC3s) represent another important source of IL-17A and IL-17F. We found that the cell numbers of RORγt^+^ ILC3s were comparable in the colon tissues of *Sarm1*^+/+^ or *Sarm1*^−/−^ mice (Fig. 6C). Also, the presence of ILC3s was not affected by pharmacologic ablation (Fig. 6F) or chemogenetic inhibition (Fig. 6H) of catecholaminergic axons. We FACS-sorted primary ILC3s from the colon tissues for the *in vitro* treatment with norepinephrine. Similar to the above observation with T_h_17 cells, norepinephrine robustly increased the levels of *IL-17A* and *IL-17F* in primary ILC3s (Fig. 6L). This norepinephrine action could be attributed to the enhanced expression of *RORγt* (Fig. 6M). These results demonstrated that the neurotransmitter norepinephrine cell-intrinsically promoted the pro-inflammatory IL-17 cytokines in T_h_17 cells and ILC3s, thus providing a mechanistic insight to the disease-mitigating function of neurodegeneration of catecholaminergic axons under the colitis condition.

## DISCUSSION

This study assessed the 3D anatomy of the ENS in the gut tissues of the mouse, non-human primate, and human. Aided with this advanced imaging power, we observed the profound neurodegeneration of catecholaminergic axons under the colitis conditions both in humans and mice. Such axonal degeneration involves the phenomenon of local energy deficit, i.e., depletion of ATP and NAD^+^ levels, and depends on the Sarm1 signal. Therefore, this unique neurodegenerative event within the ENS shares the key features of pathological axonal degeneration that our colleagues and we have documented in prior contexts (Wang et al., 2005; Gerdts et al., 2015; Yang et al., 2015; Coleman and Hoke, 2020; Figley and DiAntonio, 2020).

However, in contrast to the canonical view that axonal degeneration would contribute to disease deficits, we revealed that the neurodegeneration of catecholaminergic axons has an essential role in protecting against colon inflammation. To our knowledge, this finding represents among the first examples that a neurodegenerative event could be beneficial for a specific disease. Accordingly, it sheds light on the critical question as to why the molecular machinery designated for pathological axonal degeneration is preserved in the body. Future research might elucidate additional scenarios in which axonal degeneration acts with a disease-mitigating effect, which would advance our comprehensive understanding of the pathophysiology of neurodegeneration.

We showed that cholinergic axons within the ENS were not affected under the colitis condition. It is tempting to speculate that this selective vulnerability might be due to the differential expression of TNFα receptor, which warrants more detailed examinations. A prior study suggested that the P2X7R-mediated signal participated in the death of mouse enteric neurons during dinitrobenzene sulfonic acid-induced colitis (Gulbransen et al., 2012). Also, a recent report showed the specific loss of glutamatergic neurons in the small intestine during *Salmonella* infection via the Nlrp6/Casp11-dependent mechanism (Matheis et al., 2020). Our current work has indicated that separated molecular pathways could be in charge of different pathological processes. It will be informative to examine whether neurodegeneration within the ENS occurs in other gastrointestinal diseases such as cancers and whether it might be controlled by the alternative signaling mechanism(s).

The reciprocal neuroimmune interaction between the local inflammation and neurodegeneration in the colon is of particular interest. It has become well recognized that the nervous system can influence the body’s immunity (Webster et al., 2002; Padgett and Glaser, 2003; Glaser and Kiecolt-Glaser, 2005). For instance, the hypothalamic-pituitary-adrenal axis is activated under chronic stresses, and cortisol secreted from the adrenal cortex into the blood broadly dampens immune responses in different tissues (Glass and Ogawa, 2006; Cain and Cidlowski, 2017). Also, the autonomic nervous system can respond to stress challenges, and epinephrine and norepinephrine released by the adrenal medulla similarly exhibit immunosuppressive effects (Madden et al., 1995; Wong et al., 2012). Distinct from these classic hormonal controls of the immune system, recent studies have begun to suggest that the nervous system could modulate immune responses, including those in the gut, by more local and direct actions. For example, a previous report showed that catecholaminergic innervations in the small intestine facilitated the differentiation of muscularis macrophages to combat *Salmonella* infection (Gabanyi et al., 2016). Also, nociceptor sensory axons innervating the small intestine could instruct microfold cells to limit the bacterial invasion (Lai et al., 2020). However, whether an immune response in the gut might alter the ENS, which in return could control the inflammation, had been uncharted. Our current work demonstrated that TNFα produced during the mucosal damage triggered the neurodegeneration of catecholaminergic axons in the colon. This axonal degeneration depleted the neurotransmitter norepinephrine, which would otherwise promote T_h_17 and ILC3s to express the pro-inflammatory IL-17 cytokines. Thus, the feedback mechanism was established for limiting the colitis condition (Fig. S6). Conceptually, the catecholaminergic axons served as the “fuse” in such a neuroimmune circuit, enabling timely protection against the local inflammation. It is essential to note that norepinephrine acted as the pro-inflammatory signal in the context of our study. In contrast, norepinephrine is generally considered to be anti-inflammatory, e.g., the sympathetic signal was suggested to suppress chronic colitis in mice (Straub et al., 2008).

In sum, we revealed the pathological axonal degeneration within the ENS that has a previously-unrecognized beneficial role in colitis. This finding opens up a new dimension in our knowledge of neurodegeneration, and at the same time, exemplifies the intriguing crosstalk between the nervous system and the immune system in the gut.

## MATERIALS AND METHODS

### Non-human primate and human tissues

The non-human primate gut tissues (i.e., small intestine and colon) were collected from male rhesus macaque monkeys of 5 years old in compliance with the protocol approved by the Institutional Animal Care and Use Committee (IACUC) of the Kunming Institute of Zoology, Chinese Academy of Sciences.

The human tissues were collected in compliance with the protocol approved by the Institutional Ethics Committee of Peking University Third Hospital (IRB00006761-M2020123) and informed consent signed by all involved patients. The normal ileum and colon tissues were sampled during the right hemicolectomy for colon cancer. The colon tissues with ulcerative colitis were obtained from patients (males and females) of 24 to 75 years old.

### Mouse information

All the experimental procedures in mice were performed in compliance with the protocol approved by the Institutional Animal Care and Use Committee (IACUC) of Peking University. Mice utilized in the experiments were 8- to 10-week old females unless otherwise specified. The mice were maintained on the 12 h/12 h light/dark cycle (light period 7:00 am ~ 7:00 pm), with the standard chow diet and water available *ad libitum*. C57BL/6 wild-type mice were purchased from Charles River International. *Sarm1*^−/−^ (Cat#JAX:018069, RRID:IMSR_JAX:018069) and *Th-Cre* (Cat#JAX:008601, RRID:IMSR_JAX:008601) mice were obtained from The Jackson Laboratory, and in-house bred to produce littermates for experiments.

The *Sarm1*^*fl*/*fl*^ mouse line was generated with the targeting vector that contained the loxP sites flanking the exons 2 to 5 of the *Sarm1* gene. The linearized vector was microinjected together with the CRISPR/Cas9 system into the fertilized oocytes of C57BL/6 mice. The resulting offsprings were backcrossed with the C57BL/6 wild-type mice for two generations before further breeding.

### iDISCO(ace)

The iDISCO(ace) method was exploited, as we previously reported (Liu et al., 2020). This advanced imaging technique enabled the whole-tissue 3D assessment of diverse cellular structures, including but not limited to neuronal cell bodies/axons, blood vessels, lymphatic vessels, glial cells, and immune cells in the unsectioned gut tissues of the mouse, non-human primate, or human.

The gut tissues were freshly harvested, and digested contents were flushed out with phosphate-buffered saline (PBS). The tissues were fixed at room temperature with PBS/1% paraformaldehyde (PFA)/10% sucrose for 2 h, followed by PBS/1% PFA for 2 h. The tissues were washed with PBS at room temperature for 1 h three times. The tissues were incubated at room temperature with 25% acetone (diluted in ddH_2_O) for 1 h, 50% acetone for 3 h, and 25% acetone for 1 h. The tissues were washed at room temperature with PBS for 1 h twice and with PBS / 30% sucrose for 4 h. The tissues were decolorized with PBS/30% sucrose/1% H_2_O_2_/10 mmol/L EDTA-Na (pH 8.0) at 4 °C overnight. The tissues were washed at room temperature with PBS for 1 h twice, followed by PBS/0.2% TritonX-100/0.1% deoxycholate/10% DMSO/10 mmol/L EDTA (pH 8.0) overnight. All the incubation steps were performed with gentle rotating.

The tissues were blocked with PBS/0.2% TritonX-100/10% DMSO/5% normal donkey serum at room temperature overnight. The tissues were then immunolabeled with the intended primary antibodies (1:500 dilution) in PBS/0.1% Tween-20/10 μg/mL heparin/5% normal donkey serum at 37 °C for 72 h. The tissues were washed with PBS/0.1% Tween-20/10 μg/mL heparin at room temperature for 24 h, with the fresh buffer changed every 6 h. The tissues were further immunolabeled with the Alexa Fluor dye-conjugated secondary antibodies (Thermo Fisher Scientific, 1:500 dilution) in PBS/0.1% Tween-20/10 μg/mL heparin/5% normal donkey serum at 37 °C for 72 h. The tissues were washed with PBS/0.1% Tween-20/10 μg/mL heparin at room temperature for 48 h, with the fresh buffer changed every 8 h. All the incubation steps were performed with gentle rotating.

The immunolabeled tissues were embedded in PBS/0.8% agarose blocks before the optical-clearing steps. The tissue blocks were incubated at room temperature with 20% methanol (diluted in ddH_2_O) for 1 h twice, 40% methanol for 2 h, 60% methanol for 2 h, 80% methanol for 2 h, 100% methanol for 2 h, and 100% methanol overnight. The tissue blocks were incubated at room temperature with a mixture of dichloromethane and methanol (v:v = 2:1) for 2 h twice, followed by 100% dichloromethane for 1 h three times. The tissue blocks were finally incubated at room temperature with 100% dibenzyl-ether for 12 h twice. All the incubation steps were performed with gentle rotating.

The primary antibodies utilized for the whole-tissue immunolabeling were rabbit anti-neuronal class III β-tubulin (TUJ1; Covance, Cat#MRB-435P-100, RRID:AB_663339), rabbit anti-tyrosine hydroxylase (TH; Millipore, Cat#AB152, RRID:AB_390204), goat anti-vesicular acetylcholine transporter (VAChT; Millipore, Cat#ABN100, RRID:AB_2630394), anti-PGP9.5 (Proteintech Group, Cat#14730-1-AP, RRID:AB_2210497), rat anti-platelet endothelial cell adhesion molecule 1 (PECAM1; BD Biosciences, Cat#553370, RRID:AB_394816), goat anti-vascular endothelial growth factor receptor 3 (VEGFR3; R&D Systems, Cat#AF743, RRID:AB_355563), rat anti-lymphatic vessel endothelial hyaluronic acid receptor 1 (LYVE1; Thermo Fisher Scientific #14-0443-82, RRID:AB_1633414), rabbit anti-glial fibrillary acidic protein (GFAP; Millipore, Cat#AB5804, RRID:AB_2109645), rat anti-CD3 (BD Biosciences, Cat#555273, RRID:AB_395697), and rabbit anti-mCherry (Abcam, Cat#ab167453, RRID:AB_2571870).

### 3D lightsheet imaging

The immunolabeled and optically-cleared gut tissues were imaged on the LaVision Biotec Ultramicroscope II equipped with the 2×/ NA0.5 objective (MVPLAPO) covered with a 10 mm-working-distance dipping-cap. The tissues were immersed in the imaging chamber filled with 100% dibenzyl-ether. For imaging at 1.26× (0.63× zoom) magnification, each tissue was scanned by three combined lightsheets from the left side with a step size of 4 μm. For imaging at 6.4× (3.2× zoom) magnification, each tissue was scanned by three combined lightsheets from the left side with a step size of 2 μm. For imaging at 12.6× magnification (6.3× zoom), each tissue was scanned by a single lightsheet (middle position) from the left side with a step size of 1 μm.

Imaris (https://imaris.oxinst.com/packages) was used to reconstruct the image stacks obtained from the lightsheet imaging. Orthogonal projections were generated for representative 3D images shown in the figures. The movies were produced with a constant frame rate of 30 fps. For the display purpose in figures and movies, a gamma correction of 1.3–1.6 was applied to the raw data.

For the quantification of axons within the mouse colon tissues, the fact that the tissue thickness might change due to the colitis pathology was taken into consideration. Therefore, four 200 μm × 200 μm square areas were randomly selected along the luminal side of the mucosa in reconstructed 3D images of each colon tissue. Each square area bounded the volume through the mucosa, regardless of the layer thickness. TH-positive catecholaminergic axons or VAChT-positive cholinergic axons in each volume were manually traced.

### Mouse experiments

For the dextran sulfate sodium (DSS)-induced colitis, DSS (MP Biomedicals) was dissolved in ddH_2_O to a final concentration of 3%. The solution was 0.22-μm filtered and provided to the mice as drinking water *ad libitum*. The body weight of each mouse was followed daily before and after the DSS treatment. Also, the disease activity index of each mouse was scored according to the reported criteria (Murthy et al., 1993), which combined and averaged the severity of weight loss (0, none; 1, <5%; 2, 5%–10%; 3, 10% –15%; 4, >15%), stool consistency (0, normal; 2, loose; 4, diarrhea), and rectal bleeding (0, none; 2, bleeding; 3, bleeding >1 day; 4, bleeding >2 days).

For the treatment of anti-TNFα neutralizing antibody (Bio X Cell, Cat#BE0058, RRID:AB_1107764), the mice were intravenously injected with the anti-TNFα antibody or control IgG at 5 mg/kg of body weight. The mice were immediately subjected to the DSS-induced colitis.

For the establishment of bone-marrow chimeric mice (BMCMs), the wild-type recipient mice were irradiated. 2 × 10^6^ cells of the bone-marrow suspension from the donor mice were transplanted into each irradiated mouse via intravenous injection. At 6 weeks after the transplantation, BMCMs were subjected to the DSS-induced colitis.

For the pharmacologic ablation of catecholaminergic axons within the ENS, each wild-type mouse was administered daily with 3 mg 6-OHDA (Sigma, dissolved in 200 μL sterile PBS containing 0.1% ascorbic acid) via intraperitoneal injection for 2 days. At 2 days after the 2nd injection, the mice were subjected to the DSS-induced colitis.

For the chemogenetic inhibition of catecholaminergic axons in the ENS, the retrograde-labeling AAV viral vectors [Addgene, AAV-hSyn-DIO-hM4D(Gi)-mCherry] that expressed the chemogenetic inhibitor in the neuron-specific and Cre-dependent manner were utilized. 3-week-old *Th-Cre* or wild-type littermates were intraperitoneally injected with the AAV viral vectors (~5 × 10^10^ viral genomic copies per mouse). At 3 weeks after the viral injection, the mice were subjected to the DSS-induced colitis, with the supplement of 1 mg/mL clozapine N-oxide (CNO) to drinking water.

For the histologic examination, the tissues at the middle one-third portion of the colon were dissected out, fixed with PBS/1% PFA at room temperature overnight, and processed for the H&E (hematoxylin and eosin) staining. Five imaging areas (10× magnification) were randomly selected from the H&E-stained sections of each tissue. Histologic scoring was performed according to the reported criteria (Erben et al., 2014) with modification, which combined the severity of crypt loss (0, none; 1, <25%; 2, 25%–50%; 3, 50%–75%; 4, >75%) and immune cell infiltration (0, <5% of the lamina propria area; 1, 5%–10%; 2, 10%–25%; 3, 25%–50%; 4, >50%).

For the qPCR analysis, the tissues at the middle one-third portion of the colon were dissected out without the mesentery and flushed with PBS to remove digested contents. Total RNAs were extracted by the RNeasy Mini Kit (Qiagen) and then analyzed by the SYBR Green Real-Time PCR Kit (Thermo Fisher Scientific).

For the FACS analysis, the tissues at the middle one-third portion of the colon were dissected out without the mesentery, flushed with PBS to remove digested contents and cut into small pieces on ice. The tissues were washed at 37 °C with Hanks’ balanced salt solution (HBSS) containing 10 mmol/L HEPES/5 mmol/L EDTA (pH 8.0)/1 mmol/L DTT/3% heat-inactivated fetal bovine serum (HI-FBS; Sigma) for 20 min, followed by HBSS containing 10 mmol/L HEPES for 20 min. The tissues were then digested with RPMI-1640 (Life Technologies) containing 10 mmol/L HEPES/3% HI-FBS/10 μg/mL Liberase (Roche)/20 μg/mL DNase I (Sigma) at 37 °C for 30 min, and mashed through a 70-μm cell strainer. The resulting cells were pelleted, resuspended in Dulbecco’s modified eagle medium (DMEM, Life Technologies) containing 10 mmol/L HEPES/3% HI-FBS/40% Percoll (GE Healthcare), and purified by centrifugation through PBS/80% Percoll. The purified cells were stained with intended FACS antibodies and processed on the BD LSRFortessa. The FACS data were analyzed by FlowJo (https://www.flowjo.com).

### Norepinephrine measurement

The norepinephrine measurement was performed by the Metabolomics Facility at Technology Center for Protein Sciences of Tsinghua University. The tissues at the middle one-third portion of the colon were dissected out without the mesentery, flushed with PBS to remove digested contents, and immediately quenched with 80% methanol (diluted in ddH_2_O) pre-chilled at −80 °C. The tissues were homogenized on dry ice and stored at −80 °C for 2 h. The mixtures were then centrifuged at 4 °C for 20 min to remove the tissue debris. The supernatants were taken and dried in a Speedvac.

The samples were analyzed on the Dionex Ultimate 3000 UPLC System coupled to the TSQ Quantiva Ultra Triple-Quadrupole Mass Spectrometer with the heated electrospray ionization (HESI) probe in the positive-ion mode. The BEH amide column (Waters) was used to separate the samples. Mobile phase A: 95% acetonitrile/5% ddH_2_O/10 mmol/L ammonium formate, adjusted to pH 3.0 by formate; Mobile phase B: 50% acetonitrile/50% ddH_2_O/10 mmol/L ammonium formate, adjusted to pH 3.0 by formate; Linear gradient: 0 min, 2% B; 1.2 min, 2% B; 4.5 min, 98% B; 6 min, 98% B; 6.1 min, 2% B; and 8 min, 2% B.

The data was acquired in the selected reaction monitoring (SRM) for norepinephrine with the transition of 170/107. Both the precursor and its fragment ion were collected with a resolution of 0.7 FWHM. Spray voltage: 3,500 V; Ion transfer tube temperature: 350 °C; Vaporizer temperature: 300 °C; Sheath gas flow rate: 30 Arb; Auxiliary gas flow rate: 10 Arb; Collision-induced dissociation gas: 1.5 mTorr. Analysis and quantification were performed by Xcalibur 3.0.63 (Thermo Fisher).

### Neuronal cultures

Celiac ganglia were dissected from neonatal mice at postnatal day 3. The ganglia were incubated with 0.05% trypsin/EDTA (Life Technologies) at 37 °C for 15 min to dissociate catecholaminergic neurons. The dissociated neurons were resuspended with the Neurobasal medium [Neurobasal (Life Technologies) containing 2% B-27 supplement (Life Technologies), 2 mmol/L glutamine, 100 U/mL penicillin, 100 μg/mL streptomycin, and 50 ng/mL nerve growth factor (Sigma)]. The neurons were placed into the 24-well plates pre-coated with poly-*L*-ornithine (Sigma) and mouse laminin (Life Technologies). At 2 days after the culture setup, the neurons were changed to the Neurobasal medium supplemented with the mitotic-inhibitor mixture (5 μmol/L 5-fluoro-2’-deoxyuridine and 5 μmol/L uridine) to eliminate non-neuronal cells. At 4 days after the culture setup, the neurons were treated with indicated cytokines (R&D Systems, a final concentration of 50 ng/mL).

For the quantification of the integrity of catecholaminergic axons, the cultured catecholaminergic neurons were immunostained with rabbit anti-tyrosine hydroxylase (TH; Millipore, Cat#AB152, RRID:AB_390204), followed by the Alexa Fluor dye-conjugated secondary antibody. The immunostained axons were visualized by the epifluorescence microscope. Four imaging fields (10× magnification) were randomly selected from each well, and distal axons in each field were examined with any fragmentation as a sign of axonal degeneration. Four replicate wells were included for each experimental condition.

For the measurement of ATP levels, the ATPlite Luminescence Assay System (PerkinElmer) was utilized. In parallel, NAD^+^ levels were determined by the NAD Assay Kit (Abcam). Four replicate wells were included for each experimental condition.

### Immune cell cultures

T_h_17 cell cultures were performed according to the established protocols (Jiang et al., 2018). Briefly, the spleens of 6-week-old wild-type mice were mashed through a 70-μm cell strainer on ice. After lysing red blood cells, splenocytes were purified by the CD4^+^ T Cell Isolation Kit (Miltenyi Biotec). The resulting CD4^+^ T cells were stained with the intended FACS antibodies and sorted on the BD FACSAria to isolate naïve CD4^+^ T cells (CD4^+^CD25^−^CD62L^hi^). FACS-sorted naïve CD4^+^ T cells were suspended in the Improved Minimum Essential Medium (Life Technologies) containing 10% HI-FBS, 2 mmol/L glutamine, 1× non-essential amino acids (Life Technologies), 1 mmol/L pyruvate, 100 U/mL penicillin, 100 μg/mL streptomycin, 55 μmol/L β-mercaptoethanol, 2.5 μg/mL anti-CD28 (BD Biosciences, Cat#553295, RRID:AB_394764), 10 μg/mL anti-IL-4 (BD Biosciences, Cat#559062, RRID:AB_397187), 10 μg/mL anti-IFNγ (BD Biosciences, Cat#559065, RRID:AB_2123177), 1 ng/mL recombinant TGFβ (R&D Systems), 20 ng/mL recombinant IL-6 (R&D Systems), and 20 ng/mL recombinant IL-23 (R&D Systems). The cells were cultured in the 48-well plates pre-coated with anti-CD3e (BD Biosciences, Cat#553058, RRID:AB_394591) for the differentiation of T_h_17 cells. At 3 days after the culture setup, the cells were split at a ratio of 1:4 with the fresh medium. At 2 days after splitting, T_h_17 cells were treated with norepinephrine (Sigma, a final concentration of 10 μmol/L). Total RNAs were then extracted from the cells by the RNeasy Mini Kit and analyzed by the SYBR Green Real-Time PCR Kit.

Primary ILC3s (CD45^low^Lin^−^KLRG1^−^CD90.2^+^CD127^+^; Lin^−^: CD3^−^CD4^−^CD8a^−^TCRβ^−^TCRγδ^−^CD19^−^Gr1^−^CD11b^−^CD11c^−^NK1.1^−^TER119^−^) in the colon were FACS-sorted on the BD FACSAria according to the published method (Talbot et al., 2020). ILC3s were rested overnight in DMEM containing 20% HI-FBS, 100 U/mL penicillin, 100 μg/mL streptomycin, and 50 ng/mL recombinant IL-23 before the treatment with 10 μmol/L norepinephrine. Total RNAs were then extracted from the cells by the RNeasy Mini Kit and analyzed by the SYBR Green Real-Time PCR Kit.

### Statistical methods

Student’s *t*-test (two-tailed) or ANOVA test (one-way or two-way, with *post hoc* tests) was performed using GraphPad Prism (http://www.graphpad.com/scientific-software/prism). Statistical details of the experiments are included in the figure legends.

## Electronic supplementary material

Below is the link to the electronic supplementary material.

Supplementary material 1 (PDF 9846 kb)

Supplementary material 2 (MOV 7 kb)

Supplementary material 3 (MOV 19399 kb)
