# Supplementary material for: Sarm1-mediated neurodegeneration within the enteric nervous system protects against local inflammation of the colon
Source: Protein Cell. 2021 Apr 19;12(8):621–38. doi: 10.1007/s13238-021-00835-w (PMC8310542; doi:10.1007/s13238-021-00835-w)
Supplement: Supplementary file 1 — Supplementary material 1 (PDF 9846 kb) [file 13238_2021_835_MOESM1_ESM.pdf]

Supplemental Video Legends

Video S1. 3D assessment of the enteric nervous system within the mouse colon.

The unsectioned colon tissue of the adult mouse was processed for the whole-tissue co-immunolabeling of TH (green) and VAChT (red). The tissue was imaged at 1.26x magnification on the lightsheet microscope. 3D distribution of catecholaminergic and cholinergic innervations in the colon was shown.

Video S2. 3D assessment of the enteric nervous system and immune cells within the mouse small intestine.

The unsectioned small intestine tissue of the adult mouse was processed for the whole-tissue co-immunolabeling of TH (green), VAChT (red), and CD3 (blue). The tissue was imaged at 12.6x magnification on the lightsheet microscope. 3D distribution of catecholaminergic and cholinergic innervations and T cells in the 500-μm thickness of the small intestine was shown.

Supplemental Figures

Figure S1

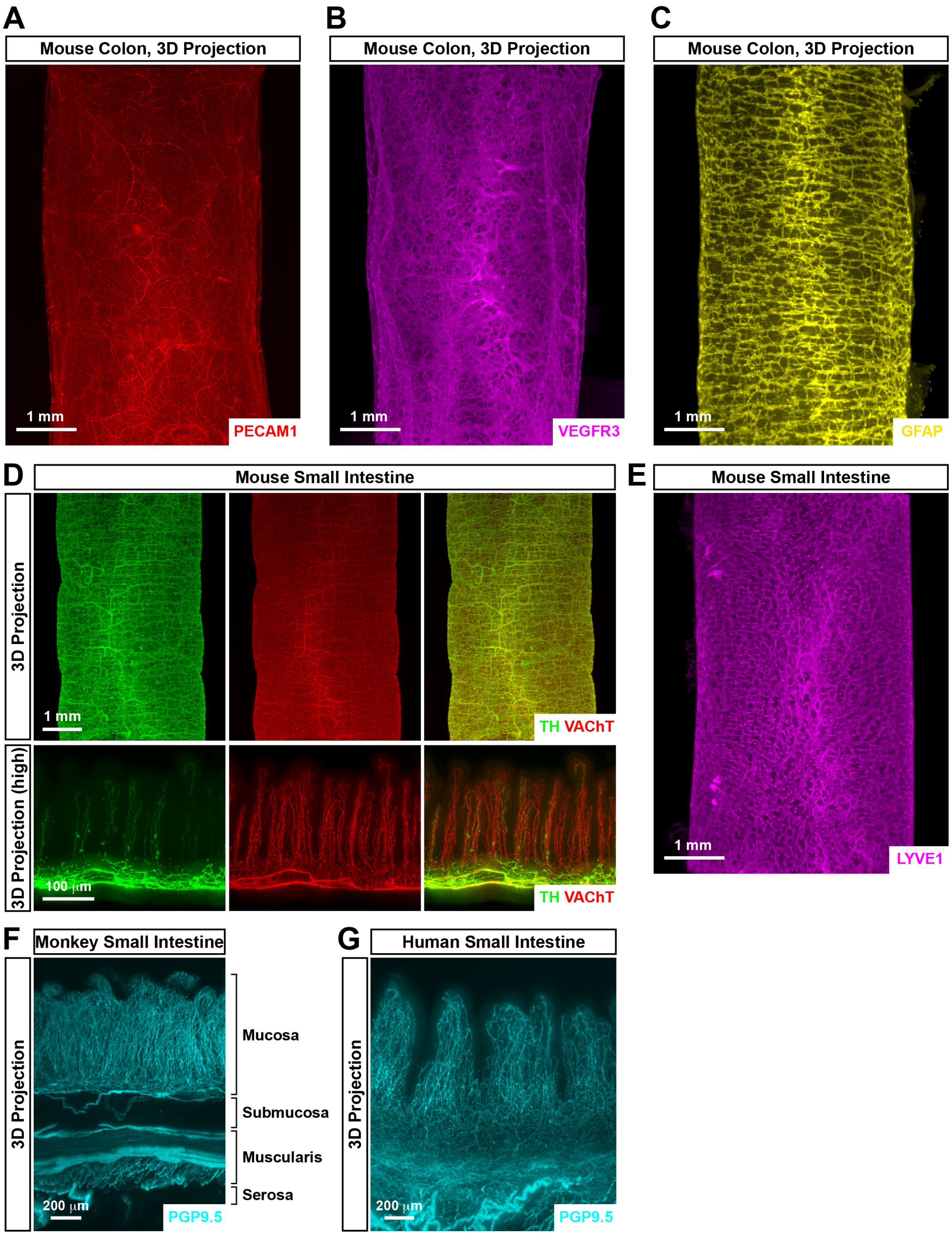

Figure S1. 3D assessment of different cellular structures within the gut tissues. **(A to C)** The unsectioned colon tissues of adult mice were processed for the whole-tissue immunolabeling of PECAM1 **(A)**, VEGFR3 **(B)**, or GFAP **(C)**. Representative 3D-projection images at 1.26x magnification of the lightsheet imaging were shown. **(D)** The unsectioned small intestine tissue of the adult mouse was processed for the whole-tissue co-immunolabeling of TH (green) and VAChT (red). Representative 3D-projection images at 1.26x magnification **(upper panels)** and 3D-projection images (longitudinal view) of the 500-μm thickness of the tissue at 12.6x magnification **(lower panels)** of the lightsheet imaging were shown. **(E)** The unsectioned small intestine tissue of the adult mouse was processed for the whole-tissue LYVE1-immunolabeling. A representative 3D-projection image at 1.26x magnification of the lightsheet imaging was shown. **(F)** The unsectioned small intestine tissue of the adult macaque monkey was processed for the whole-tissue PGP9.5-immunolabeling. A representative 3D-projection image (cross-sectional view) of the 2-mm thickness of the tissue at 6.4x magnification of the lightsheet imaging was shown. The anatomical layers (mucosa, submucosa, muscularis, and serosa) were indicated. **(G)** The unsectioned small intestine tissue of the adult human was processed for the whole-tissue PGP9.5-immunolabeling. A representative 3D-projection image (cross-sectional view) of the 1-mm thickness of the mucosa at 12.6x magnification of the lightsheet imaging was shown.

Figure S2

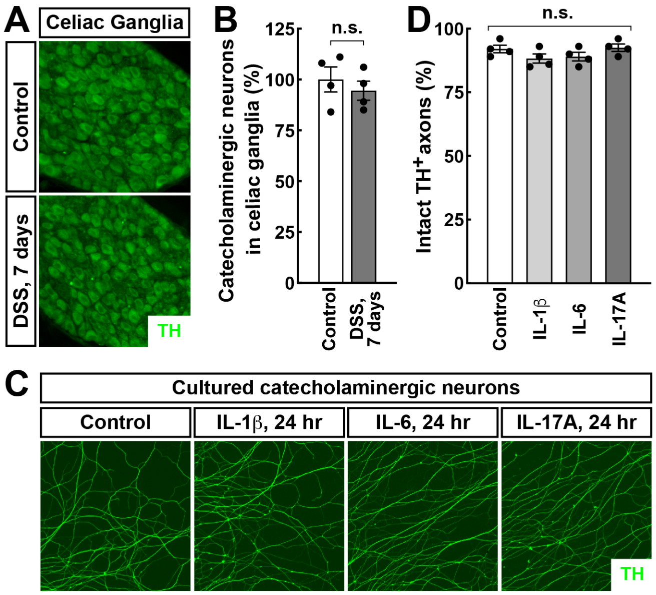

Figure S2. Catecholaminergic neurons of the celiac ganglia in the DSS-induced colitis.

(A and B) The wild-type mice were subjected to the DSS-induced colitis. The celiac ganglia of the mice were then processed for conventional immunohistochemistry. **(A)** Representative images of TH-positive catecholaminergic neurons in the celiac ganglia. **(B)** Catecholaminergic neurons in the celiac ganglia were quantified. n = 4, mean ± SEM, n.s., not significant (Student's *t*-test). **(C and D)** Catecholaminergic neurons of the celiac ganglia of wild-type mice were *in vitro* cultured and treated with each indicated cytokine (final concentration of 50 ng/ml). **(C)** Representative images of TH-positive axons of the cultured catecholaminergic neurons. **(D)** The integrity of TH-positive catecholaminergic axons was quantified. n = 4, mean ± SEM, n.s., not significant (ANOVA test).

Figure S3

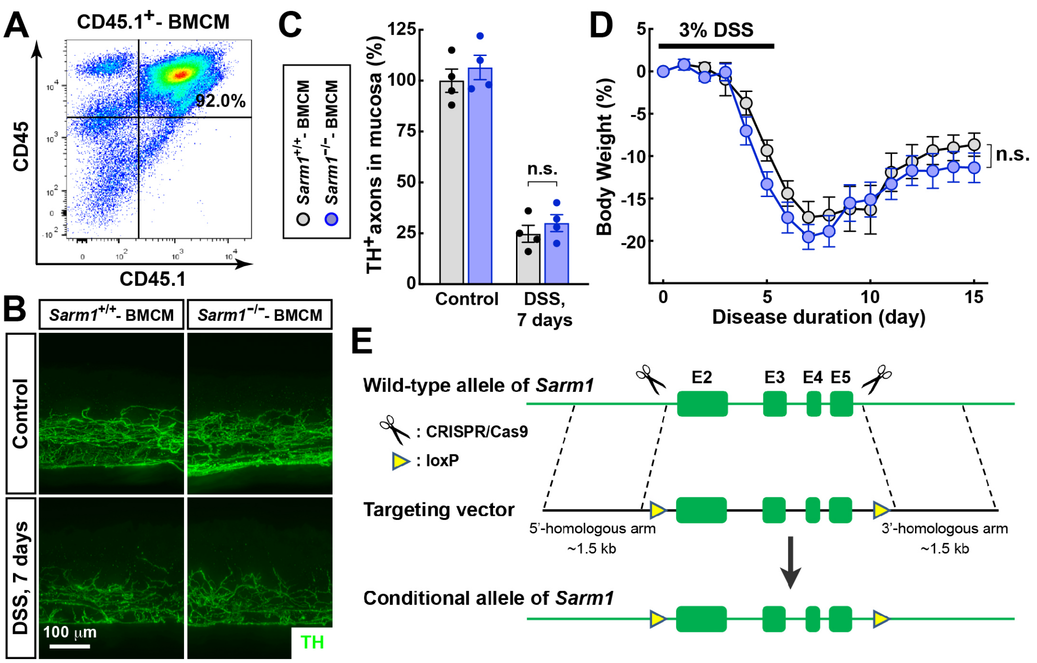

Figure S3. The *Sarm1* depletion in immune cells did not affect the DSS-induced colitis.

(A) The wild-type recipient mice of CD45.2$^+$ background were irradiated and transplanted with the bone marrow of CD45.1$^+$ donor mice. The chimeric efficiency of CD45.1$^+$-BMCM (bone-marrow chimeric mice) was examined by the FACS analysis of immune cells in the spleen. **(B to D)** The wild-type recipient mice were irradiated and transplanted with the bone marrow of *Sarm1*$^{+/+}$ or *Sarm1*$^{-/-}$ donor mice. *Sarm1*$^{+/+}$-BMCM and *Sarm1*$^{-/-}$-BMCM were then subjected to the DSS-induced colitis. **(B)** The unsectioned colon tissues were processed for the whole-tissue TH-immunolabeling. Representative 3D-projection images (longitudinal view) of the 500-μm thickness of the tissues at 12.6x magnification of the lightsheet imaging were shown. **(C)** TH-positive catecholaminergic axons within the mucosa were quantified. n = 4, mean ± SEM, n.s., not significant (ANOVA test). **(D)** The body weights of the mice were followed through the colitis condition. n = 8, mean ± SEM, n.s., not significant (ANOVA test). **(E)** Diagram of generating the conditional allele of *Sarm1*.

Figure S4

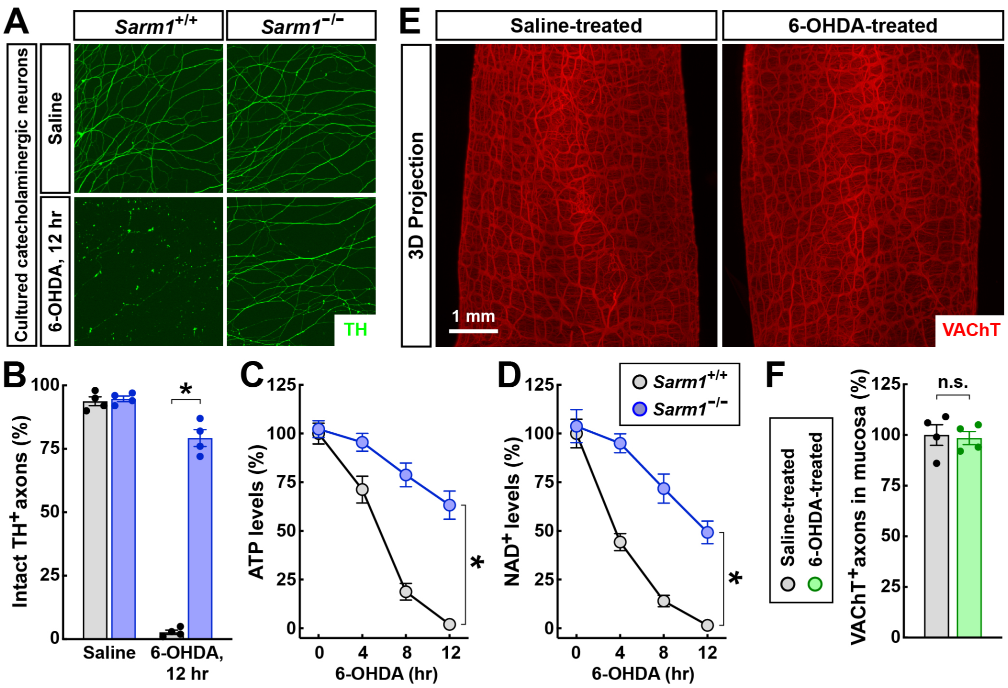

Figure S4. Pharmacologically-induced axonal degeneration of catecholaminergic neurons of the celiac ganglia.

(A to D) Catecholaminergic neurons of the celiac ganglia of *Sarm1*$^{+/+}$ or *Sarm1*$^{-/-}$ mice were *in vitro* cultured and treated with 50 µM 6-OHDA. **(A)** Representative images of TH-positive axons of the cultured catecholaminergic neurons. **(B)** The integrity of TH-positive catecholaminergic axons was quantified. n = 4, mean ± SEM, * $p < 0.01$ (ANOVA test). **(C and D)** ATP **(C)** or NAD$^+$ **(D)** levels of the catecholaminergic neurons were measured. n = 4, mean ± SEM, * $p < 0.01$ (ANOVA test). **(E and F)** The wild-type mice were treated with 6-OHDA via intraperitoneal injection. The unsectioned colon tissues were then processed for the whole-tissue VAChT-immunolabeling. **(E)** Representative 3D-projection images at 1.26x magnification of the lightsheet imaging were shown. **(F)** VAChT-positive cholinergic axons within the mucosa were quantified. n = 4, mean ± SEM, n.s., not significant (Student's *t*-test).

Figure S5

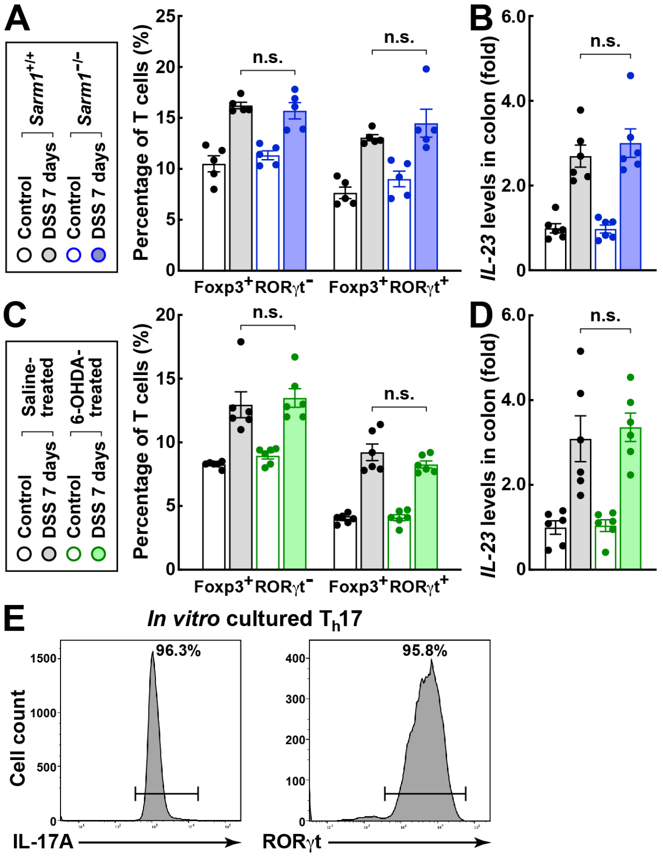

Figure S5. Manipulation of the catecholaminergic axons in the DSS-induced colitis. (A and B) *Sarm1*$^{+/+}$ or *Sarm1*$^{-/-}$ mice were subjected to the DSS-induced colitis. **(A)** Foxp3^{+} RORγt^{-} or Foxp3^{+} RORγt^{+} T cells in the colon tissues were FACS analyzed. n = 5, mean ± SEM, n.s., not significant (ANOVA test). **(B)** *IL-23* mRNA levels in the colon tissues were determined. n = 6, mean ± SEM, n.s., not significant (ANOVA test). **(C and D)** The wild-type mice were treated with 6-OHDA via intraperitoneal injection and then subjected to the DSS-induced colitis. **(C)** Foxp3^{+} RORγt^{-} or Foxp3^{+} RORγt^{+} T cells in the colon tissues were FACS analyzed. n = 6, mean ± SEM, n.s., not significant (ANOVA test). **(D)** *IL-23* mRNA levels in the colon tissues were determined. n = 6, mean ± SEM, n.s., not significant (ANOVA test). **(E)** *In vitro* cultured T$_h$17 cells were FACS analyzed for the protein expression of IL-17A or RORγt.

Figure S6

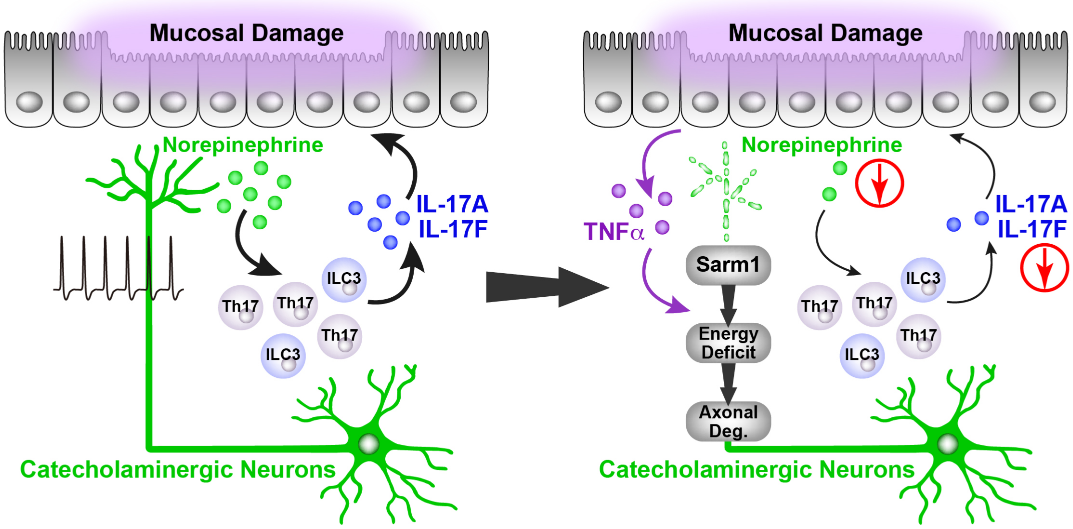

Figure S6. Sarm1-mediated neurodegeneration of catecholaminergic axons protects against local inflammation of the colon.
